# Autistic discussion forums: insights into the topics that clinicians don’t know about

**DOI:** 10.3389/fpsyt.2023.1271841

**Published:** 2023-12-19

**Authors:** Catherine L. Caldwell-Harris, Tiffany McGlowan, Katherine Beitia

**Affiliations:** Department of Psychological and Brain Sciences, Boston University, Boston, MA, United States

**Keywords:** autism, user forums, content analysis, online spaces, diagnosis, myths and facts

## Abstract

**Background:**

User-led autism discussion forums provide a wealth of information about autistic lived experiences, albeit oriented toward those who regularly use computers. We contend that healthcare professionals should read autism discussion forums to gain insight, be informed, and in some cases, to correct assumptions about autistic persons’ lives and possibilities. But experts may be dismissive of user-led forums, believing forums to be filled with myths, misinformation, and combative postings. The questions motivating our research were: Do online forums raise issues that are educational for clinicians and other stakeholders? Are forums useful for those who do empirical research?

**Method:**

Content analysis was conducted on 300 posts (62,000 words) from Reddit, Quora, and Wrong Planet. Forums were sampled to reflect broad topics; posts were selected sequentially from the identified forums. The authors read through posts in the Excel sheet, highlighting statements that were the main ideas of the post, to discern both broad categories of topics and more specific topics. We coded content pertinent to classic autism myths and analyzed attitudes towards myths such as ‘lack emotion’ and ‘cannot form relationships.’ To document whether forum posts discuss topics that are not widely known outside of elite experts, we compared discussion content to new material about autism contained in the March 2022 DSM 5 Text revision.

**Results:**

Classic autism myths were discussed with examples of when elements of myths may be valid. Posters described cases where parents or therapists believed myths. Experts may believe autism myths due to rapid changes in diagnostic practices and due to their lack of knowledge regarding the characteristics of autistic people who have typical intellectual abilities. We conclude that forums contain high-value information for clinicians because all concepts in the DSM 5 text revision were discussed by posters in the years before the text revision appeared. Ideas that are only slowly becoming part of the research literature are discussed at length in forums. Reading and analyzing forums is useful for both clinicians and scientists. In addition, the relative ease of forum analysis lowers the bar for entry into the research process.

## Introduction

Health-related online discussion forums have grown in popularity as people learn from each other while sharing daily life struggles (1). How should experts in the field of autism, such as clinicians, therapists, educators, and researchers, view autism discussion forums? Forums are widely appreciated for giving voice to autistic people (2, 3). Online communities share discourses about how to develop an autistic identity, understand autism as a culture, and reframe deficit as difference (4). Discussion forums also provide practical implications for friends and family members (3–5). But are forums relevant to experts? Autistic people have long expressed frustration that autism is misunderstood (6, 7), as in this quote reported by Linton et al. (3): *That’s why I say that ‘experts’ frequently do not get autism at all. They truly need our input. However, I also realize that they do not generally consider us a reliable source.* Consistent with this quote, researchers have argued that autistic people should be viewed as autism experts (8–10). The current paper examines whether online discussion forums provide relevant, timely, and insightful information not just for lay people but for experts.

Change in how autism is viewed has been rapid. Diagnosis has evolved, with growing numbers of people diagnosed in adulthood (6). With changes in diagnostic criteria, the majority of autistic people do not have an intellectual disability (11, 12). Current criteria appear to be male-centered leaving women undiagnosed (3, 13, 14). Autistic people are encouraged to seek employment, leisure activities, and fulfillment in life commensurate with their abilities (7, 15–18). Some research analyzes forums to characterize autistic perspectives (3, 4) but there is currently no systematic examination of whether the content of autistic forums is likely to be novel and revelatory for researchers and clinicians.

### Analyzing forum content with quantitative and qualitative methods

Forum analysis refers to extracting information, both quantitative and qualitative, from online discussion forums. Both quantitative and qualitative analyses can be applied to discussion posts drawing on the techniques for analyzing any textual information (19). A quantitative approach is to count the frequencies of words or phrases; and interpret their significance (20). Qualitative approaches involve identifying consistent themes across several texts (21). We briefly review prior examples of forum analysis.

Giles and Newbold (1) examined the history of online self-help networks and online communities where people discuss their experiences of diverse psychiatric disorders. They discussed ethical considerations, methods for selecting forums, and analytical techniques. Using DSM-IV-related search terms, those authors constructed a large corpus of forum posts. Their specific research question was how users discussed the importance of having an official diagnosis. Healthcare professionals have traditionally been cautious about the value of user-led forums, as this can encourage self-diagnosis. Somewhat surprisingly, many users demonstrated a reverence for having an official diagnosis (1). Formal diagnoses allowed users to present themselves as part of an in-group with special authority to speak about their disorder or disability.

Interestingly, a later content analysis of autism discussion posts found a different attitude towards diagnosis. Sarrett (22) analyzed posts on Wrong Planet, a website designed to celebrate autism in all its diversity, hosting diverse forums as well as articles and videos about employment, schools, and parenting. Sarrett categorized comments according to their primary attitude: accept self-diagnosis, reject self-diagnosis, and middle-of-the-road. Those who rejected self-diagnosis were concerned about legitimacy, like the reports in Giles and Newbold (1). However, arguments for accepting self-diagnosis were plentiful (23) and focused on the difficulty of obtaining a diagnosis as an adult, skepticism about diagnostic practices, and being a self-expert.

Another noteworthy example of forum analysis examined Facebook groups for caregivers of Alzheimer patients. The authors used a careful longitudinal method to detect patterns in daily-life situations over a 6-month period. Caregivers most frequently discussed their exhaustion, desire to give up, and struggles in communicating with and obtaining help from family members, such as sharing care responsibilities. Another category was discussion of violent behavior by people with Alzheimer’s dementia, a topic understudied by researchers.

Discussion forums have been analyzed for how they are used in positive and prosocial ways. This is particularly true of autism forums, as the internet has allowed the emergence of a vibrant autism community. Internet forums allow autistic individuals to connect with others not just for social support, but to organize and advocate for recognition of autistic cultural differences (2). An example is Parsloe’s (4) analysis of The Aspies Central Website. She documented how users participated in and contributed to a worldwide shift in the understanding of autism from a biomedical to a cultural perspective. Forums have also been used as a source of data for basic science questions, including autistic person’s religious beliefs (24) and special interests (25). A survey that asked autistic people to report their religious beliefs yielded similar results to quantitative coding of discussion forums where autistic forum users discussed their religious beliefs (24).

### Dangers of discussion forums: misinformation, myths, and combative exchanges?

For autism discussion forums to be touted as a source of insight for clinicians, we must address the popular perception that social media sites spread fake news, false information, and rumors (5). Discussion forums allow large amounts of unverified information to spread rapidly (26), making this a concern in health-related fields (27). Autism myths have been featured for years in books and informational websites (28). Surveys reveal that both autistic and non-autistic people have uneven knowledge about autism (9, 29). If myths are discussed in online communities, it is important to learn if false information is shared. If myths are rebutted in online discussions, it would be useful to analyze whether the discussion about myths goes beyond information commonly known to experts.

Experts seeking insights will lack the motivation to read user-led forums if they have an argumentative or nasty tone, as has been reported for some online sites (30). Incivility has been noticed most strongly in political discussion forums, but anonymity on the internet could license sarcasm and mockery in a variety of online forums. Rösner et al. (31) conducted an online experiment in which participants read a news article about marijuana legalization, followed by between zero and six uncivil comments. Exposure to even one comment with a hostile tone was sufficient to increase readers’ negative cognitions. It is thus worthwhile to determine how often uncivil comments appear in autistic online discussion forums.

### Motivation and overview of the current article

Our goal was to address whether forums are useful for clinicians, educators, and researchers. Our own reading of forums, including our prior forum analysis, suggested forums had potential as rich sources of insight, even for experts. But experts may be wary of user-led forums, given popular concerns that social media is filled with myths, misinformation and combative postings (5). Our first goal was to scrutinize posts to determine how often they contained misinformation and myths, and if present, how did other posters respond to these. We also wanted to establish whether discussion forums can provide insight to experts such as clinicians (9). Establishing whether posts are informative to experts has a subjective element since this depends on those experts’ knowledge base and experience. What material could substantiate a subjective impression that discussion posts discuss up-to-the-minute issues? We tackled this by comparing discussion topics to newly revised material in the March 2022 DSM-5 Text Revision. If online forums, with material dated prior to March 2022, review the topics in this revision, it would lead credence to the claim that discussion forums frequently contain current, informative information.

## Method

### Selecting platforms, forums and posts

Reddit, Quora, and WrongPlanet were used as these are publicly accessible social media platforms (websites) with written postings in a question-answer format. Wrongplanet is a website serving the autistic community. Reddit and Quora are large websites that host a variety of forums focusing on specific topics, although the topic of a forum (usually identified by its title) can range from general to specific in content (see examples in Table 1). Our team was already familiar with the forums dedicated to autism on Reddit and Quora, from prior extensive forum analysis (32). We already knew the titles of many of the specific discussion forums that had been created by autistic individuals for sharing and information exchange for other autistic people. Because new forums can be created and old ones retired, we began by using the search terms *Reddit autism*, *Quora adults with autism, Quora autism in women.* A target of 300 posts was set as a number large enough to extract generalizations but small enough to be tractable. Table 1 lists the 15 forums selected, number of posts within each forum, and other relevant information, including examples of questions.

**Table 1:** Characteristics of posts selected with examples of questions.

	**Number of posts**	**Average words per post**	**Number of threads**	**Example of the question starting a thread**
**Quora (8 forums)**				
Autistic girls and women	50	142	16	How to get my parents to show me my autism diagnosis papers?
Exploring autism	20	254	8	Did you struggle with impostor syndrome after your autism diagnosis?
Autism awareness	20	245	3	Are people with high functioning autism more similar to neurotypical people than to people with low functioning autism?
Autistic people matter	15	170	9	Do autists have good intuition? it goes a bit against their rational, logical nature?
Asperger syndrome	15	424	2	Is it common for Aspies to suddenly withdraw from relationships?
High functioning autism	10	485	8	Do people treat you like you are an idiot because of your autism diagnosis even if you are actually smart?
Living with autism	5	425	4	Has Aspergers made employment difficult?
Autistic nerds and geeks	5	583	3	When should I tell my child they are on the spectrum?
				*Total Quora posts: 140*
**WrongPlanet (3 forums)**				
Women’s discussion	25	131	11	Was Diagnosis a Life Changer?
Adult autism issues	25	215	18	Am I on the asexual scale because I’ve had relationships without wanting to have sex?
In-depth adult life discussion	10	330	7	Can autism be conditioned out of a person by corrective behavior?
				*WrongPlanet posts: 60*
**Reddit (4 forums)**				
Ask autistics	50	132	6	I have always had difficulties relating to romance in media.
Aspergers	25	104	2	I stopped masking, now i do not even remember how to.
Autism	20	269	17	I was diagnosed with Autism as a child but parents hid it from me. I found out when I was 19.
Autistic adults	5	57	4	Does anyone else get bothered by how empathetic they are?
				*Reddit posts: 100*
Total forums = 15		Total words in corpus: 62,000 Total posts: 300		

The researchers who selected forums and discussion questions were the two co-authors (TM and KB), who were students in the early days of the project, and 3 additional undergraduate lab members. The corpus of 300 posts was compiled before any hypotheses were made and before any decisions had been made about content to investigate. The goal was to compile a broad set of posts and discover the topics. The student-led team coordinated with each other on selecting forums and how to paste posts into an Excel spreadsheet for later analysis. The group agreed to select forums on highly general topics, since the goal at the beginning of the project was to compile forums covering broad, general topics. Forums devoted to broad, general topics are also the most frequently accessed and perused by users, so these are forums that are preferentially delivered in response to search queries. A corpus of broad, general topics will also resemble what ordinary internet users might experience if they decided to explore autism discussion forums. To convey to readers the meaning of “general topics,” all of the forums used are listed in Table 1. Examples of more specific forums that were avoided are, from Reddit: Spicy Autism, Autistic With ADHD, and Autism Translated.

For each forum selected, team members agreed to start with the first discussion thread that they saw on the website, and to paste into the Excel sheet additional posts in the thread until the discussion thread was concluded, or until between 10 and 25 posts had been obtained from a selected forum. Posts were excluded if they had under 30 words or were clearly off-topic. Our corpus of posts was constructed in the spring of 2021 and reflected posts from November 2017 to April 2021. Posts from prior years were included because searches in web browsers, as well as Quora and Reddit algorithms, preferentially present posts that are highly accessed and highly up-voted.

It is typical to think of discussion forums as being short exchanges between numerous participants, mimicking a conversation. However, our corpus contained many lengthy posts which presented (or responded to) a unique question. These “one-off” posts are common at Quora. Quora specializes in short questions (usually just one sentence) which are then answered in-depth by a person with expertise in the topic. Answers are voted up by readers, leading to a single high-quality post being presented prominently on the Quora website.

#### Ethical concerns

The institutional review board at our university did not consider forum analysis to be human subjects research, because our team did not interact with any persons online. This stance is consistent with other research on forums (1, 33). Nonetheless, this is a grey area and norms may be changing. Some researchers post an introductory note to inform users that academic researchers are reading posts for research purposes (22). However, this practice infuses researchers’ goals into the forum, potentially disrupting a space for autistic-only voices, and could load an additional set of expectations onto posters (34).

### Content analysis

#### Broad topics

The authors read posts in the Excel sheet to identify what topics were being discussed. Like conventional content analysis (19), we grouped topics into categories and sub-categories. The authors discussed these categories during lab meetings and ultimately agreed on the presence of 10 broad topics. Categories, subcategories, and their frequencies are listed in Supplementary Table S1, available in the supplemental information. We also coded whether posters reported having an official diagnosis (22%), being self-identified (43%), being a parent (7%), not being autistic (2%), being neurodivergent (2%) or providing insufficient information to categorize (24%). The posts that contained no information had content consistent with identifying as autistic. Note that none of the posts discussed in the section on myths or DSM-5 relevance were made by parents or by people who identified as not autistic.

After conducting a content analysis of topics, our team had long discussions about the insightful ideas described in the corpus. It was at that point that we turned our attention to the idea of analyzing the 300 posts to address whether forums are useful for clinicians, educators, and researchers. The posts were not selected in advance to reference myths, or to reference topics discussed in the DSM 5 text revision.

#### Myths and misinformation

To determine whether forums contained any disinformation, we analyzed the corpus for references to myths about autism. Focusing on myths has the advantage that many websites exist which list myths in straightforward language. These websites also explain why these are myths and what facts are accepted by experts. We used the search term *autism myths* to identify lists of myths. We picked 2 websites due to their high scientific prestige: the Kennedy Krieger Institute, and the US Department of Health and Human Services. We included a third website, Autism Awareness,^1^ due to its focus on education, its non-profit status, and because it was a reputable source outside of the US. Each website contained 5–8 myths, which we amalgamated together to create a master list of 17 myths either about autism or about autistic people (see Table 2). When we describe the myths, we retain the language *people with autism* as this terminology is used in the sources from which we derived the myths.

- *Six myths about autism*: Autism is a disease, is relatively new, can be cured, can be cured by special diets, is caused by vaccines, is caused by bad parenting.
- *Eleven myths about autistic persons*: Autistic people are generally or always nonverbal, savants, violent, lack emotions, lack empathy, have mental/intellectual disabilities, cannot stand to be touched, cannot learn, cannot form relationships, have no sense of humor, do best at jobs which entail repetitive tasks.

**Table 2:** No posts endorsed myths but posters made three types of comments.

Total posts in corpus of 300	Myth	Elements of myth valid	Others believe myth	Challenge myth	Percentages (out of 52 posts with myths)
10	Lack empathy	5	1	4	19
10	Lack emotion	5	1	4	19
6	Cannot stand to be touched	6	0	0	12
6	Cannot form relationships	1	2	3	12
4	No sense of humor	3	1	0	8
4	Have intellectual disabilities	2	0	2	8
4	Best at repetitive tasks	0	0	4	8
2	Is a disease	0	0	2	4
2	Can be cured	0	2	0	4
2	All are nonverbal	0	2	0	4
1	Poor parenting	0	0	1	2
1	Autism is relatively new	0	0	1	2
0	Cured by special diets	0	0	0	0
0	Caused by vaccines	0	0	0	0
0	Cannot learn	0	0	0	0
0	Are violent	0	0	0	0
0	Are savants	0	0	0	0
Total 52	Total	22	9	21	

Combining three sources created a more comprehensive list than using a single source, and was consistent with statements used to measure knowledge about autism in surveys by Gillespie-Lynch et al. (9) and Stone (35). Also pertinent are 10 myths in the book *Life on the autism spectrum: Translating myths and misconceptions into positive futures* (28). Their 10 myths were all in our set except Bennett et al. (28) included the myth of the autism epidemic. Bennet et al. (28) included refrigerator mothers while our list of myths referenced the more general category ‘bad parenting.’

All posts were scrutinized for the presence of these myths or for anything else that seemed medically incorrect or non-standard. We also noted whether any posts contained hurtful comments or a generally angry or negative tone.

#### Comparing forum content to novel material on autism in DSM 5 text revision

We wanted to rigorously examine the impression that autistic people posting on discussion forms frequently have insights about autism that are not widely known to clinicians and researchers, thus demonstrating that autistic people sometimes have considerable expertise regarding autism, in addition to insider knowledge. To be representative of autistic interests and concerns, our corpus contained a variety of topics. We thus instead sought to demonstrate that autistic discussion forums contained a diversity of knowledge about autism that was not widely known to typical autistic experts.

## Results

We first briefly address our broad analysis of the content of posts, before turning to the specific questions guiding the project.

### Broad categories, topics, and sub-topics

Topics are categories summarizing content, whereas themes are more abstract concepts that include higher-level goals, feelings, and attitudes. We did not have any specific hypotheses about what topics would be discussed in forums. To provide context for our analyses of myths and the DSM 5 text revision, we briefly discuss content analysis of topics. Supplementary Table S1 lists the most common topics identified in the corpus. These are not surprising. Prior peer reviewers informed our team that documenting these topics did not constitute a contribution to the research literature, but we included them as Supplementary material for interested readers.

The most frequently discussed category of topics was *Autism as a Mental Health Condition*. The most common topics within this broad category were the diagnostic process, male/female differences, and sensory sensitivities. A second broad category was *Social Interaction*, where the most frequent topics were difficulty interacting with others, and relationship advice. Our third broad category was *Challenges*, which included the topics of achieving well-being and parenting, such as advocating for school services or learning about one’s autism following a child’s diagnosis. Our final category of topics was *Interests and Talents*, with the most common topics being special interests and artistic abilities. Special interests were dominated by entertainment and intellectual pursuits, such as psychology, science, and gender studies. Quotes for several topics were extracted to illustrate the quality and variety of posts; these appear in the Appendix (Supplemental materials).

In the next sections, we organize our results under subheadings corresponding to our main questions:

- Did posts contain misinformation and myths?
- Were posts angry or combative?
- Could forums provide insights for healthcare professionals?

### Did posts contain misinformation and myths?

None of the posts advocated for the validity of autism myths, nor was medical misinformation found in the posts. Of the 300 posts, 52 posts made reference to at least some aspect of the classic autism myths, although in most cases the myth itself was not mentioned. None of the posts mentioned more than one myth (because the purpose of posts was not to discuss autism myths; another topic was foregrounded). We coded posts that referenced aspects of myths into the following three categories, corresponding to what seemed to be posters’ purpose.

- to refute or challenge myths
- to discuss the ramifications of other people incorrectly believing myths.
- to discuss how elements of myths could be valid in some cases or could apply to themselves or could apply in restricted situations.

The quotes excerpted below cover all cases of each myth discussed in our corpus (with exceptions noted below).

#### Myth: autism can be cured

Two posts discussed other people who apparently believed this myth.

- *My partner once mentioned that he used to be autistic, but his real dad put him through conditioning to stop his autism from being an issue…* [continued in subsequent post after another post asked for details] *His real dad used to put loud static on headphones and force him to listen to it for hours at a time to reduce his sound sensory. He do not talk to his real dad anymore.*
- *Course now with what’s happened with his grief and epilepsy his autistic traits are showing more often though his mum still claims he’s cured and the traits are merely cause he’s mirroring me.*

#### Myth: autism is caused by poor parenting

While no post endorsed this myth, one thread opened with: *Do you think there’s a connection between autism and overprotective parents?* The eight responses rejected the idea of a causal link, as in this example: *Are you asking if overprotective parenting causes autism? If so, the answer ranges from No to Extremely Unlikely. Are you asking if autism causes overprotective parenting? If so, the answer ranges from Possible to Very Likely* (challenge myth).

Three of the 8 responses noted that overprotection could make their autistic characteristics worse. The most explicit example was: *....it could make children less self-reliant, take opportunities away to practice social skills, prevent them from gaining life experience, reduce self-esteem, cause social anxiety and hamper the development of resilience, thereby making autistic symptoms more noticeable or making the transition to adulthood and independence harder.* These responses could be seen as ‘elements of myths could be valid’ (because a parenting practice was claimed to exacerbate autistic symptoms), but we decided not to code these responses in this way. Our reasoning was that the posts primarily concerned young adults complaining about parental over-protection. Parental over-protection is distinct in meaning from ‘poor parenting’ and “making autistic symptoms more noticeable” is distant from “causing autism.”

#### Myth: people with autism are non-verbal

[Discussion with psychiatrist] *She told me that she no longer thought I needed to see a Neurologist and that people with autism are nonverbal and that I did not seem to be one of those kinds of people* (other people believing myths).

One poster described how her good verbal ability caused her mother to disbelieve her autism diagnosis. *My mother will not even read my autism diagnosis. She constantly compares me with people she works with* [in adult social care]*, saying I do not understand, they cannot even speak.... I think she is in denial* (other people believing myths).

Three posts referenced atypical speaking manner, which our team evaluated as distinct from being nonverbal and thus not endorsing elements of the nonverbal myth. These posts are nonetheless interesting as examples of autistic posters’ concerns.

- *I was raised in Hong Kong, where no one knew about Aspergers 40 years ago, I was always being punished by acting and speaking not normal.*
- *I struggle with my tone in voice. I speak too fast or too slow. Too loud or too soft.*
- *He can seem “neurotypical” to those who do not know him because he looks “normal” and speaks “normally.” Until he does not.*

This next quote probably meant “speaking rarely” rather than being nonspeaking: *I got away with not speaking because girls are allowed to be “shy.”*

#### Myth: autistic people lack emotion

Posts discussed many commonplace aspects of human emotional life. The word *emotion* appeared 37 times in the 62,000-word corpus. Six posts discussed different facets of reduced levels of emotion. We thus labelled these as consistent with ‘elements of myths could be valid in some cases.’ We grouped the 6 posts into the following four categories.

- Not understanding emotion. *Not understanding your own emotions is a trait of autism, Emotional Intelligence is hard...*
- Not identifying emotions. *I cannot really decipher facial expressions. I am bad at reading emotions of others. (And my own). This quote includes a mix of statements about emotion. I feel emotions very deeply but have a hard time identifying what they are. And I can feel other people’s emotions but also have trouble identifying them and what not. It’s especially hard when they cry because I kinda just sit and stare and try to think of what to say and cannot so I end up looking like a jerk.*
- Not discussing emotions. *Deep emotional conversations about how much you mean to me are not the norm for aspies. We aren’t good at explaining how we feel, either physically or emotionally.*
- Emotional shutdown. *Complete shutdowns when afraid or sad*. A second post combined over-emotion, dysregulation and shut-down: *Why you have meltdowns and either lose your shit or just totally shut down.*

The following reports described having too much emotion, which goes against the myth. We thus included these as cases of ‘challenging myth.’

- Emotion dysregulation. *Why you cannot control your emotions sometimes and you hate yourself for it*. Two additional posts also described dysregulation.
- Absorbing others’ emotions (relates to empathy, see below). *…emotional ‘sponge’ (I would absorb others’ feelings when they were sad or angry, thus acting like I was)*
- Social expectations for women to be emotional. *[It]costs all women a lot to be social and emotional shielding for everyone else, but when we are autistic the costs rise brutally fast.*

#### Myth: autistic people lack empathy

One poster recommended that non-autistic people should *... try to meet any autistic people without assumption of delayed emotions, or lack of empathy* (other people believing myths).

In discussing her own symptoms, one poster noted that her problem with empathy was *not so much issues with feeling empathy, but with expressing it.* Four other posts made similar statements (elements of myths could be valid in some cases).

In contradiction to the myth, three posters discussed having too much empathy.

*A life of being taken advantage of, because you are so empathetic, even if you do not know how to show it, and you feel EVERYTHING. A life begging to know why you are different. Why you do not fit in.*

*Does anyone else get bothered by how empathetic they are? I’ve always been naturally empathetic and compassionate and not until I was around 19 did I really start to be able to turn that off. I still cannot control my emotions a lot but I’ve gotten a good enough handle on my empathy where I can tune into it if I want to or ignore it (usually).*

*While talking about how we feel can be hard, a lot of us are strangely empathetic. Like, painfully empathetic.*

One poster argued that autistic people are natural empaths. *We spend our whole lives adapting to what people think we should be. We are human chameleons. In essence, we are empathic metamorphs (from Star Trek - Next Generation - The Perfect Mate episode), which is a being that can sense what people around her desire and react accordingly. It is pretty telling that at age 22, I identified heavily with this character. One often misunderstood trait of autistic, is our internal empathic accuracy. This means we make great social psychologists, or that we are great at predicting the thoughts and feelings of another person—we are actually better than non-autistics at this.*

#### Myth: autistic people cannot stand to be touched

Three posters mentioned dislike of being touched. One poster stated this about herself in response to a post asking people to share their autistic symptoms. *I hate being touched. Especially skin-to-skin contact.* A second post mentioned that “*avoids being touched by others” is a common sign of autism.* A third post responded to a request for signs that someone is autistic. The list of signs included: *dislike of touch, does not touch others and/or avoids being touched by others, sometimes physically moving the other person’s hand to another part of the body (forearm instead of hand).*

Three additional posts discussed sensory sensitivity to things touching their skin, such as *wearing clothes inside-out so that seams will not be touching my skin*. One post conveyed the feeling to readers: *Imagine you are sunburnt from head to toe. Any touch is absolute torture. Then you have to wear that itchy, scratchy sweater that grandma knitted you last Christmas*. One post noted the impact on a spouse*: I am sensitive to texture and touch all over, it can be an issue with a touch oriented spouse. He is mindful of my sensitivities and I know he needs touch emotionally so we try to compromise.*

The six posts described here were examples of how elements of myths could be valid in some cases. Eight additional posts discussed sensory sensitivities but were not specific to being touched.

#### Myth: autistic people cannot learn

No post referenced this myth. The posts that came closest to it were two posts about being too tired or not motivated sufficiently to learn. One concerned how cognitive abilities may be altered due to stress associated with autism. *Most of us with autism are so stressed that our learning, memory, digestion, and healing circuits are closed.*

As would be expected in any vibrant and earnest discussion of human development, the corpus contained varied and complex references to learning, with 76 mentions of ‘*learn’* (and *learned, learning*). Example included: *meltdowns can appear at a young age...as a child gets a bit older they might learn that outward expressions of their distress are ‘bad’...* We did not code these remarks as challenging the myth, because the statements referenced learning as what all humans do, with no implications for autism. An exception to this was a post that implicitly challenged the myth by noting the opposite of ‘cannot learn’: autistic learn quickly when interested*. Autists are very sensitive and learn very fast if we are interested in something* (challenge myths).

One post mentioned enjoyment in learning on one’s own. *I learn new things on my own. Languages and information that I find to be interesting. I love to learn new things, and I believe that I have studied psychology and attachment styles on the same level as someone who chose such a path in terms of education. I speak 7 languages, and 6 are self-taught.*

#### Myth: autistic people cannot form relationships

One poster specifically condemned clinicians for believing this myth: *I had an assessment and was told I cannot be autistic because I’m married. Many women I know have been denied the diagnosis based solely on their ability to maintain some relationships* (other people believing myths).

One poster obliquely referenced the myth, while denying it, by writing: *But never pretend that a person who is autistic does not contribute hugely in a relationship* (challenge myth).

Posters discussed the difficulty of making friends: *Making platonic friends is something I find incredibly difficult*. Discussion also included challenges in romantic relationships: *Funnily my partner had the same problems; we are both autistic, both ADHD, and both started the relationship with rejection issues*. One poster referenced an extreme level of difficulty: *It’s a life of loneliness and relationship problems you do not understand* (elements of myths could be valid in some cases).

The above quotes were the only ones that touched on autistic people have difficulties with relationships. Discussion of relationships were prominent across many different threads, figuring in 1 out of 6 posts, while the word *relationship* occurred 77 times in the corpus of 300 posts. Examples included: *How long into a relationship do I wait to tell my partner I’m autistic?* And: *Give the person more time and perhaps take a step back in your relationship* (*especially your physical relationship*). These rich discussions implicitly challenge the myth that autistic people cannot form relationships, but we did not tally them in Table 2 as they are typical for ordinary human conversation and did not intersect with statements about autism.

#### Myth: people with autism have no sense of humor

Three posters mentioned difficulty understanding jokes, which we coded as ‘elements of myths could be valid in some cases.’

An autistic-identified woman wrote *when I make a joke,* [my autistic husband] *can think I am criticizing him.* A second poster mentioned struggling with jokes along with other aspects of language: *...as a girl I was pretty good at masking and only struggled with a few things such as figures of speech or certain jokes and especially rhetorical questions like what’s the point of asking if you do not want an answer.* A third poster mentioned not understanding jokes in the same context of not liking other NT activities. *Do people treat you like you are an idiot because of your diagnosis even if you are actually smart? ... until I bust out with something pertinent to their discussion. Or some scientific gobbledygook that amazes them. I do not get some jokes, and I do not follow sports or watch much tv.*

*School was a living hell for me and I’ve always been seen as the ‘slow one’ or the ‘handicapped kid who did not get jokes’* (other people believing myths).

#### Myth: autistic people do best at jobs that entail repetitive tasks

One post asked: *What kind of jobs do you have and what advice do you have about jobs for autistic people?* A second asked: *Why cannot some people with Aspergers hold paid jobs?*

Many posts discussed interview difficulties, social expectations, masking and job stress as reasons for not having a full-time job. No post in our corpus mentioned repetitive tasks or even a structured routine as a benefit. Four posts argued against structure and/or the drawback of conformity and job boredom. We thus coded these as ‘challenge myth.’

- *I hate schedules with a passion, if you mean having a day heavily planned in advance. I find that I do well with one scheduled meaningful activity about every other day, and I build the rest of my activities around that as the mood strikes me.*
- *I managed to attend college and I’ve had plenty of jobs – so I’ve “managed” to fit myself into rigorous timetables but I think it’s against my nature to work mere 9 h days. Having to work the same hours, everyday – every week – it seems like slow death by monotony to me.*
- *I would start out very excited to learn something new. Once I mastered the job, which did not take long, I would become bored.*
- *Employment has been a really hard road for me, I’ve spent so many years smashing up neurotypical social niceties, like I was driving a crazy clown car…. But if you want to join the monkey troop, you are going to have to make some compromises.*

#### Myth: autism is a disease

*Autism is not a disease, it is simply a different way that some people’s minds process the world around them* (challenge myth).

*You do not have autism, it is not a disease or mental illness. In order to avoid the stigma for both of you, say you are autistic* (challenge myth).

#### Myth: all autistic people have mental/intellectual disabilities

Two posts discussed the general category of autism without intellectual disability, classified a ‘challenge myth.’

- *Impostor syndrome is rampant ...that they are faking it... particularly prevalent in autism without intellectual disability* (challenge myth).
- *Until recently, autistic females without an intellectual disability were often misdiagnosed or overlooked* (challenge myth).

One post discussed autistic individuals with mental/intellectual disabilities.

- *My family does not want to understand my Autism, mental disabilities, and mental illness at all* (elements of myth could be valid).

How different types of disabilities intersect with autism was a frequent topic. The words *disabled*, *disability* and *disabilities* occurred 43 times in the corpus. Posters discussed what disabilities co-occurred with autism, whether autism is a disability, and topics related to the social mode of disability. They also discussed practical concerns such as insurance coverage, disclosing one’s disabilities in the workplace and to friends, and general coping strategies.

#### Myths not discussed in the corpus

Five remaining myths were not referenced in our corpus. No posters mentioned special diets to ameliorate symptoms, although posts did mention sensory issues with food texture, digestive problems, and being picky about food choices. No posters said autism was relatively new or linked autism with vaccines, savants, or violence.

### Were posts angry or combative?

In none of the posts did users mock, derogate, or criticize other posts. This relieves a concern raised by prior researchers (30, 31). On occasion, posters politely expressed their disagreements on specific topics. To illustrate the high level of civility we saw on the forums, we list here the strongest cases of disagreement in our corpus.

*I disagree with those who say the only early intervention is ABA. There’s also Floortime, which you have discovered.*

Autistics: what is your opinion on the sentence ‘everyone has a little bit of autism’? I happen to disagree with a lot of people about whether there is such a thing as a little bit of autism. At times in my life I was accused of being a little autistic because, well, I *was* a little bit autistic... [post goes on to explain more for 200 words.]

*I absolutely disagree with those saying to get a doctor to give you an official diagnosis: it’s not usually covered by insurance, it’s expensive, most cannot diagnose adults, even fewer can diagnose females...* [post continues with additional details for 100 words].

### Could forums provide insights for healthcare professionals?

The corpus contained many posts illustrating the material that was new in DSM-5-TR. Illustrative quotes are listed in the righthand panel of Table 3, corresponding to each new DSM-5-TR concept. For example, in the first row, a new item in the DSM-5-TR was “More subtle overall deficits for individuals without intellectual or language impairments.” This corresponds to growing awareness autism may be an appropriate diagnosis even without the obvious “classic” symptoms of eye contact, odd body posture, and atypical voice qualities (36). The quote concerns about preferring information-rich discussion while disliking gossip. This is “subtle” because this characteristic is not diagnostic of autism and occurs among non-autistic persons. Yet it can be seen as an impairment since the poster goes on to describe some distress, inviting the inference that this discomfort with gossip can at times impair a daily activity. The doctor referred to in row 2 was not familiar with evolving concepts of autism, since he did not know that an atypical communication style may go unnoticed in someone with overall good communication. Indeed, across the corpus of posts, distress and indignation at misdiagnosis or not being sufficiently impaired for a diagnosis was a frequent topic (see Table 1). The DSM-5-TR idea of less obvious restricted interests is also illustrated in our listing of common special interests from the corpus, in the final rows of Table 1, such as TV shows, psychology, and yoga.

**Table 3:** Comparing new concepts in DSM 5 TR to forum-analysis posts.

	**Items excerpted from DSM-5-TR, in order of appearance in that document**	**Quotes from the corpus of 300 forum posts indicating a similar concern or issue**
1	More subtle overall deficits for individuals without intellectual or language impairments	I’m exhausted by people and find talking to other women IRL particularly taxing because I hate socializing. I can carry out a conversation full of information or on an interesting topic, but if someone starts shit talking about their lover I just want to run headlong into a wall and die. *[This is an example of a subtle deficit because the posters’ exhaustion may not be obvious to observers.]*
2	More subtle deficits in social communication for individual with better overall communication skills	I was not formally tested as, after a chat with me, the doctor said because I could hold a conversation I could not be autistic. I waited a year for that appointment.. *[The poster understands that her social deficits were more subtle than not being able to hold a conversation]*
3	Less obvious restricted patterns of behavior and interests if the interests are closer to age-typical norms	Most of my special interests are in *video games*, with collecting plushies, *cards, games* themselves and what not. Another one of them is car crashes... I do not know why I am so interested in them but I’m interested nonetheless. Also *animals [interests resemble those for NTs]*
4	Stress may result from consciously calculating what most individuals find socially intuitive	I spend the whole conversation *focused on what I’m supposed to say or do* and often cannot participate because I can only focus on that
5	Lower ascertainment of autism spectrum disorder due to less obvious symptoms in these [competent] individuals, perhaps especially in adult women.	What a mess unpicking all the damage health professionals have done. Gaslighting in the worst possible way. They convinced me I was just a crazy female and it was my fault I wasn’t getting better because I wasn’t trying. Even a spell in a psych ward. Not one male health professional ever considered ASD. I was way too smart, must just be manipulative like a typical BPD. Cruelty towards me cause the stigma of BPD.
6	Stress of social interaction may be exhausting; unable to concentrate because of the mental effort in monitoring social conventions	Interacting with my Neurotypical (NT) friends, is exhausting. I have to do what is expected (i.e., my duty as a friend). I have to appear happy, sad or angry, match my emotions with the group, at the same time help them solve a problem they are facing.. prefer interacting with Aspies ... does not exhaust me.
7	Self-esteem is adversely affected by being unable to be themselves	[Diagnosis] showed me that many of my issues were not caused by character flaws and being a weak person but I was born this way. That was a great help to my self esteem. It helped me understand myself both in knowing my strengths and weaknesses. They helped me avoid situations that are not good for me and seek situations that are good for me
8	Normal theory-of-mind [ToM deficits not present in all cases].	Like many women with autism, people and social rules are one of my special interests.
9	Executive function deficits are also common but not specific, as are difficulties with central coherence [Infer: executive function difficulties common but not invariably present.]	I’m extremely slow in making artwork. I love doing it, but I insist on getting good results, and that means taking a lot of time, experimenting on it.. Could the slowness be caused by poor fine motor skills, requiring extra caution? *[slowness may indicate an atypical form of executive dysfunction or atypical weak central coherence -- or something else]*	10	Living independently and working… able to find a niche that matches their special interests and skills and thus are productively employed.	I’m 38 now and I’ve been working for a professional services firm in what’s now a well paid job for 11 years... The clincian said I wasn’t autistic because I had a job and was living independently and was in a relationship….
**Female characteristics**
11	In comparison with males with autism spectrum disorder females are likely to have…. [pertinent to 5 items below]	I wasn’t diagnosed until adulthood. Autism often presents differently in women than in men. I think a lot of people have this idea of what autism looks like that is based on a “lower-functioning” man.
12	►Better reciprocal conversation.	In general, people like to talk about themselves, and they enjoy feeling as though others are listening.. that’s part of how I’m perceived; people think I’m a good listener because I repeat back key things they say, and offer insight.
13	►More likely to share interests, to integrate verbal and nonverbal behavior, and to modify their behavior by situation, despite having similar social understanding difficulties as males	Yes, it is generally thought that autistic women are somewhat different from autistic men, and the differences overall make female presentation more subtle. But it’s not that all autistic females have the exact same traits and all autistic males have the exact same traits. Traits will still vary on an individual basis and there may be some cross-over/overlap.
14	►Masking. Attempting to hide or mask autistic behavior (e.g., by copying the dress, voice, and manner of socially successful women) may also make diagnosis harder in some females.	Masking does not mean to act as if I am NT. Masking means to learn the skills that for others are intuitive. It’s a superpower many of us here have. It comes easier to female aspergers than to male ones because our symptoms are different. Be proud of what you achieved. It does not mean hiding, it means overcoming by hard work.. NTs get gifted with what we have to learn. But we are better at learning things.
15	►Repetitive behaviors less evident	My stims are small -- small repetitive movements? (Foot/toe curls). I am easily overstimulated by light and noise
16	►Special interests may have a more social (e.g., a singer, an actor) or normative focus (e.g., horses)	I have always had special interests but because they are what you would consider “mainstream” or typical for a girl it also went under the radar.
17	Rates of gender variance increased in autism spectrum disorder, with higher variance in females compared with males	*[posed question]* How do I know if a woman has Aspergers/autism? What is the most common sign? *[response]* She was a tomboy or is gender fluid.

Row 6 describes the new idea that the stress of social interaction may be exhausting and contribute to social impairment. Six posts describe the stress of social interaction in ways similar to the description in the DSM 5 text revision. Several referred to *social exhaustion*, a term used in online neurodivergent and therapy communities. For example, one thread opened with the question: *Do you experience less social exhaustion with a person overtime?* Responses to this thread shared experiences or provided advice, such as this one: *I know that I can actually crave spending lots of time with someone who I do not feel social exhaustion with – it’s a rare experience, and thus valued highly. That being said, many autistic people need alone time to recharge or deal with sensory overload regardless.* Another post connected stress to ABA therapy (Applied Behavioral Analysis): *If she was raised with ABA she might just always be on high alert trying to read signals and react appropriately and it becomes an EXHAUSTING reaction.*

Row 7 lists the new idea in DSM-5-TR that theory-of-mind deficits are not present in all cases. The quote here provides insight into a phenomenon that is only starting to become known to clinicians: many autistic people with typical intellectual ability become good mind readers because they understand its necessity and apply themselves, and/or because understanding social rules becomes a special interest (37). The bottom section of Table 3 lists DSM-5-TR’s overview of how autism may affect females differently from males. This topic has had a long life on discussion forums (3, 14). Consistent with this, one-fifth of posts in our corpus discussed or mentioned female-specific symptoms.

*When forum posters are more knowledgeable about autism than some mental health professionals.*

In the prior section, we reviewed posts that illustrated novel material in DSM-5-TR, although they were posted between 1 and 4 years prior to the publication of DSM-5-TR. Many other posts touched on aspects that are more subtle and detailed than what is contained in the DSM-5-TR yet ring true in terms of being consistent with other autistic writings such as autie-biographies (38, 39), autistic-authored blogs (40) and the authors’ own knowledge of autistic people. We discuss 4 of these below.

#### Sobbing as a reaction to social or environmental stress

Misinterpreting crying as a sign of depression. *The one time I did go to a professional who could diagnose, the doctor told me I was depressed and gave me some Zoloft because I had a crying meltdown after she asked me about something that would obviously make me emotional (I am a little bit depressed but it’s like the very bottom of the list of things I need to worry about).*

#### Asking for help

A theme the authors have frequently encountered on autistic discussion forums concerns the difficulty of asking for help. It likely makes sense to clinicians that asking for help will be challenging for persons with social difficulties, but clinicians may not have details about this challenge, nor recognize it as consistent with autism. Nor has this difficulty been discussed systematically in the research literature (see Discussion). For this reason, we cite this as a case where people on forums are more knowledgeable than mental health professionals. One of the quotes in our database explains why asking for help is challenging.

Question*: Do Aspies find it difficult to ask for or accept offered help and favours?*

Answer*: This was something that caused me a lot of trouble my entire life. It breaks down into a few problems. ....initiating social interaction... ....the chance of them actually helping me in the way I need is lower than the chances of me solving the problem without them or them making the problem worse if I ask. ....will see me as a burden if I ask for something. ...I’m still not super awesome at asking when I need something*. [Many useful details omitted for brevity.]

#### Bothered by illogical group dynamics; ethical high standards, politically idealistic

*Not conforming to the hivemind/groupthink and seeing that the emperor has no clothes; being bothered by illogical group dynamics and detesting rude people (they are rude, because they think you are below them, while I think social hierarchies are usually not based on truths).*

*...Is overly strong-willed and can be demanding in her idealism….*

*We are loyal, we will not have an affair, or leave the other person.*

#### Sensitivity and emotional intensity

The historic association of autism with lack of emotional responsiveness has made it hard for non-autistic people, including experts, to understand that ‘highly sensitive person’, and high emotionality, are common in autism. The following discussions were in our corpus.

*Has intensity that makes her overly sensitive and relentless.*

*Over the years I have been diagnosed with depression, anxiety, and an eating disorder. I have been told I’m just “being sensitive” and that I do not “seem autistic.”*

*Autistic people are highly sensitive, and so may bruise easily, causing them to close up from others to protect themselves.*

## Discussion

No consensus exists among healthcare professionals or researchers about the value of online forums as sources of insight for professionals or as sources of data for researchers. We sought to remediate this gap in the literature for autism forums. Here we discuss the evidence that forums are useful for clinicians, educators, and researchers.

### Discussion forums contain high-value information: evidence from the analysis of myths

Concerns about misinformation have been raised in prior research (27, 30). Form analysis of autism forums revealed the opposite: the content of these forums had minimal hostility and virtually no misinformation. Consistent with prior writings about the value of online communities (3, 4), family members, friends, and autistic individuals should feel confident that browsing and contributing to autism forums will be rewarding.

Our initial goal in searching for myths in the corpus was to substantiate and qualify our prior impression that autistic forums do not spread misinformation. During analysis of how aspects of myths were discussed, we became impressed with posters’ knowledge about autism. Posts incorporated the “truth” section explained in the myth vs. truth sections of authoritative sources such as (9, 28). But the posts went beyond those by providing compelling examples that challenged myths but also dissected how elements of the myths could be valid in some cases.

### Challenging myths

Posters frequently challenged myths by sharing examples that were the opposite of the myth. Key examples were being an emotional sponge, disliking the structure, and lacking learning opportunities in the workplace. Researchers could systematically address how often traits classically associated with autism manifest as both hypo- and hyper variants [see (41)]. This a relatively novel idea and mostly confined to research on sensory sensitivity (42), although scholars have discussed hypo- vs. hyper-arousal underlying social functioning (43). Bimodality in functioning is routinely discussed in autism discussion forums, in domains barely touched by scholars, such as language, face recognition, analytical intelligence, and memory.

### Other people believe myths

Posters shared the distress, disappointment, and rejection experienced when other people, such as a parent, believed myths. Most striking of these concerned clinicians believing myths. We have seen no scholarship on clinicians endorsing autism myths or making diagnoses based on myths. Research on knowledge of autism has only been conducted on students and the general public (9, 29).

Clinicians may believe myths because of the enduring power of classic or profound autism, which is stereotyped as including traits such as “…impaired reciprocity, quality of eye contact, atypical vocal prosody, presence of motor mannerisms, and atypical gait or posture” [(36), p. 653]. Clinicians estimated such traits to occur in 40% of the autistic population, in the survey by De Marchena and Miller (36). Some respondents provided estimates as high as 90%. This survey indicates that some clinicians lack experience with the diversity of characteristics of autistic people, consistent with complaints in the current corpus about incorrect diagnoses. Future research can explore what proportion of clinicians believe myths and what this implies for diagnosis and intervention.

### Elements of myths could be true in some cases

Posters discussed how elements of myths could be true in some situations. Consider posts explaining why autistic people may be perceived by others as lacking emotions. One post noted *We aren’t good at explaining how we feel*. A second post explained that emotional shut-down is a coping strategy during emotional dysregulation. These explanations situate autistic people as similar to NTs, because NTs can suffer emotional dysregulation and may have difficulty explaining how they feel. In contrast, *lacks emotion* is alien and suggests a discontinuity with human experience.

Another nuanced treatment concerned dislike of being touched. The myth is widely held by the general public, as mentioned in a focus group conducted by John et al. [(29), p.14]: *the mother was adamant that the boy could not be autistic because he liked hugs*. The popular myth of autistic people not liking human touch may be conflated with social introversion to imply dislike of people (29). In contrast, in our corpus, posters attributed discomfort with human touch to sensory sensitivities. These thoughtful reports of touch sensitivity are pertinent to current scholarship since the role of touch-aversion in social interaction has broad implications for parenting and intervention (44).

### Discussion forums as sources of insight for clinicians

Reading online forums can provide clinicians with rapid insight into the concerns of autistic individuals (33). This is important because posters provided examples of how their misdiagnosis occurred because professionals were not aware of autistic symptoms. The ‘subtle deficits’ included in the March 2022 DSM 5 text revision have been discussed in discussion forums for years (3, 45). We documented this by comparing novel elements of the DSM-5-TR to the topics in forums. All new points in the DSM-5-TR were discussed in the corpus of 300 posts.

The DSM-5-TR item in row 15 was curious to us. Women were viewed as having a set of favorable social abilities yet were still said to have “similar social understanding difficulties as males.” This similarity remains an empirical question and one that is not supported by the quotes in our corpus discussing women’s social abilities. “Similar social understanding as males” is at odds with the comments that clinicians decide not to diagnose women because their social abilities were too competent.

Consistent with the above, a common complaint in the autism community is clinicians not recognizing symptoms of autism in adult women (3, 4, 46). Although such symptoms are increasingly discussed by scholars (6, 47), forum posters complain that these details are unknown to some clinicians. For example, one poster observed that because she had a crying meltdown during her diagnostic interview, the clinician concluded she was depressed and prescribed Zoloft. A clinician could interpret a crying episode as a sign of depression if clinicians do not know that autistic women commonly report crying meltdowns due to the stress of being questioned by a doctor in a high-stakes assessment. Also helpful is awareness that a crying meltdown need not be florid but can manifest as quiet sobbing. Here we see forum posters discussing concepts in a more detailed manner than the partial attempt in the text revision. Posters reported crying meltdowns due to stressful social interactions, such as experiencing disapproval from authority figures, or being overwhelmed by sensory stimuli, as can happen in a crowded supermarket.

Emotional dysregulation is frequently discussed in the autism community as a neurodivergent characteristic, and has attracted the attention of scholars (48), but it is not a symptom in the DSM-5 definition of autism spectrum disorders. The DSM-5-TR does state that social interaction can be stressful and exhausting (row 6 in Table 3) but does not alert clinicians that emotional dysregulation can result. This raises the question of why emotional dysregulation is not noted as a common symptom in the DSM-5-TR. Is this omission because of the continuing prominence of the stereotype (or, myth) among medical professionals that autistic people are not emotional? The unfortunate result is that clinicians regard emotional dysregulation as pointing to anxiety, depression, or borderline personality rather than autism.

### Discussion forums can inform diagnostic practices

The corpus contained many examples of topics that can extend the boundaries of professionals’ knowledge about autism. Consider the difficulty in asking for help. How to teach autistic adults to ask for help is part of social skills training (49). Autistic individuals’ reluctance to seek help has been noted when giving advice to police and has been identified in studies of the experiences of autistic college students (15, 17), and late-diagnosed autistic women (50). However, in each of the articles just cited, asking for help was included in a list of other challenges, and was not the focus of systematic exploration. Clinicians may not realize that asking for help is challenging for neurotypical-passing autistic adults.

One of the quotes in our database discussed why asking for help is challenging (see Results). When this quote was discussed with autistic acquaintances of the authors, one woman mentioned that her therapist was mystified by her difficulty in asking for help, which included making requests more broadly, such as asking her parents to buy needed items or asking for a raise at work. This therapist reportedly spent weeks in therapy trying to identify the traumatic event that prevented her client from making requests. Clinicians would benefit from knowing how asking for help requires navigating several social domains.

### Forums can provide data relevant to research questions

Empirical research suggests autistic people are less selfish than neurotypical people (51), and value abstract moral rules over social expectations (52). These journal articles managed to cast their findings as consistent with autistic deficits, rather than traits to be admired. Little scholarly work exists on autistic people’s preference for honesty, equality, and social justice, but these topics and their lived experiences are common in autism forums. A related example of a topic that pushes against the boundary of professional knowledge is autistic people’s avoidance of hierarchical group dynamics. The social activist Greta Thunberg has remarked that her autism allowed her to care less about conforming to social norms [see (53)].

Our analysis suggests an abundance of material relevant to research questions.

Researchers can analyze the content of discussion forums using qualitative methods, like analyzing free responses in questionnaires. We list here specific topics from our corpus that have been insufficiently studied by researchers but are frequently discussed in online forums (see details in Supplementary Table S1 and Supplementary Appendix).

- The experience of receiving a diagnosis: what went wrong; were tests and observations appropriate for the client’s age and intellectual abilities (3).
- The experience of disclosing one’s diagnosis vs. masking: how are decisions made about ‘pretending to be normal’ (or disclosing) and what are the ramifications (54).
- Childhood experiences of either diagnosed or undiagnosed autism: how was one treated by family, friends, teachers and others?
- Life experiences and concerns of parents who realize they are also probably autistic once their child is diagnosed.
- Understanding the impact of psychiatric conditions that statistically co-occur with autism, such as ADHD, OCD, social anxiety, and anorexia.
- Understanding the impact of co-occurring symptoms such as motor clumsiness, poor face recognition, gut problems, irritable bowel syndrome, gender fluidity, and homosexuality.
- Handling diverse social experiences such as friendships, peer pressure, bullying, dating, marriage, parenting, school and work.
- The ongoing controversy over how to integrate autism without intellectual disability compared to classic or profound autism.
- Autistic characteristics often manifest at either the low or high-end of ability; many of these are discussed in forums although this topic rarely appears in the research literature

### Limitations: the merits and disadvantages of forums

A primary limitation concerns the novelty of forum analysis as a research method. Forum analysis deserves to be a tool in researchers’ toolbox, alongside interviews and surveys (34). Each method brings its strengths and weaknesses; those of forum analysis are summarized in Table 4. For example, the strengths and weaknesses of a convenience sample vs. a random sample are well-known. Consider the challenge of how to sample online forum posts, when there may be virtually unlimited content (55), or very rare content can be sought out with sophisticated data scraping techniques. Guidelines for analyzing frequent vs. rare content have not been rigorously presented or defended. A lesson from the current project is that a representative vs. semi-random sample of forum posts cannot be undertaken without considerable familiarity with the structure of the platforms to be analyzed, including how topics are offered up to browsers when using search tools.

**Table 4:** Strengths and challenges of forum analysis as a research tool.

	Analyzing user-led discussion forums rather than surveys/interviews	
	*Strengths*	*Drawbacks and challenges*
Whose Agenda	The questions being answered are generated by forum users, not led by the researcher’s agenda	Questions on forums may only partially overlap with researchers’ questions
Demand Qualities	No researcher-induced demand characteristics	Posters’ goals in writing on a forum can influence content
Amount of Data	Depending on the topic, large or even inlimited data	There may be no data if posters do not address the topic of interest to a researcher
Demographic	Posters choose to reveal what they decide is pertinent in their post or personal profile.	Insufficient details about background characteristics
Ease	Forums easy to find online	Need to learn about specific digital cultures
Payment	Funds not needed to pay participants	Posters may appreciate their ideas informing research; or may not appreciate that quotes are used in research; Researcher does not know which is the case.
Ethics	Ethics approval may not be required (gray area)	

Our corpus included a relatively small number of posts discussing DSM-5 diagnostic practices. This detracts from conclusion validity regarding the content relevant to myths and the DSM. But our primary purpose wasn’t to analyze how autistic people discuss myths or diagnoses. Instead, our intent was to document whether myths are rare in forums. Content analysis revealed references to myths are uncommon, being mentioned in only 52 of 300 posts (17%). What is new, and mostly unknown in the research literature, is that when elements of myths were mentioned, discussion was insightful and nuanced.

The small number of posts relevant to DSM 5 is also a limitation. After being impressed with posters’ insights, we decided to analyze the corpus for discussion of topics in the DSM-5-TR. Posters did insightfully discuss topics relevant to diagnosis. This is evidence that forums autistic posts can be instructive for clinicians. This sets an agenda for future work: obtain a larger, systematically constructed corpus by identifying relevant threads and/or posts via keyword searches.

Forum data does not provide researchers the same assurance as interview or survey data, where all participants have been systematically recruited to have diagnoses [or at least self-diagnosed; see (34)]. But forum analysis is not thereby inferior to traditional surveys and interviews. Surveys and interviews are oriented around researchers’ agendas, have demand characteristics, and often have unknown self-selection biases (see Table 4). Many people participate in interviews for money. Online surveys that pay out money can be infected by AI bots whose free-response answers can be surprisingly sophisticated. One solution is to combine survey methods and forum analysis [e.g., (24), who investigated autistic people’s religious beliefs].

Researchers might be wary of forum analysis because it may seem like a shortcut. Humans value outcomes requiring large effort [effort justification effect, (56)]. But the ease of forum analysis is a feature, not a bug. When readers experience skepticism while reading a forum analysis, they can take immediate action by going to the named forums (or similar ones). Readers can use the search terms provided in the method and determine for themselves whether the content is consistent with the authors’ report.

### Autism advocacy

We have established that autistic people have substantial expertise on autism, thus going one step beyond the important paper by Gillespie-Lynch et al. (9). The current article also showcased the benefits of forum analysis. Reading and learning from forums is an accessible route for non-experts and experts alike to learn about autistics who use social media, both for insights and as a source of empirical data (34, 55). Analyzing forums does not substitute for meeting autistic people, attending conferences created by autistic people, and collaborating with them as researchers (10, 57) but forums have a low bar for entry. They are accessible at no cost to anyone with internet access who can use a computer, an advantage for researchers without labs and grant funding. Despite being a low bar, forums are rich enough to reward scholars and researchers at any level of expertise.

Forums are especially rich with voices of women, as is apparent in the current corpus in forums specifically for autistic women, and themes in the posts (see Appendix). Sexism in society is evident when accomplished males like Elon Musk are commonly regarded as both autistic and highly intelligent, yet doctors tell women they are too smart to be autistic and therefore must have BPD (as in the illustrative quote in row 5, Table 3).

Autistic persons’ positive attributes have been undervalued (12, 58, 59). So too with discussion forums. Let both prosper.

## Data availability statement

The original contributions presented in the study are included in the article/Supplementary material, further inquiries can be directed to the corresponding author.

## Ethics statement

Ethical approval was not required for the study involving human data in accordance with the local legislation and institutional requirements. The social media data was accessed and analyzed in accordance with the platform’s terms of use and all relevant institutional/national regulations.

## Author contributions

CC-H: Conceptualization, Data curation, Formal analysis, Methodology, Project administration, Resources, Supervision, Writing – original draft, Writing – review & editing. TM: Conceptualization, Data curation, Writing – original draft. KB: Conceptualization, Data curation, Writing – original draft.

## Footnotes

^1^autismawareness.com.au/
